# A Thermophilic Phage Endolysin Fusion to a *Clostridium perfringens*-Specific Cell Wall Binding Domain Creates an Anti-Clostridium Antimicrobial with Improved Thermostability

**DOI:** 10.3390/v7062758

**Published:** 2015-06-12

**Authors:** Steven M. Swift, Bruce S. Seal, Johnna K. Garrish, Brian B. Oakley, Kelli Hiett, Hung-Yueh Yeh, Rebekah Woolsey, Kathleen M. Schegg, John Eric Line, David M. Donovan

**Affiliations:** 1 Animal Biosciences and Biotechnology Laboratory, Beltsville Agricultural Research Center, Agricultural Research Service, U.S. Department of Agriculture, 10300 Baltimore Avenue, Bldg. 230, BARC-East, Beltsville, MD 20705, USA; E-Mail: steven.swift@ars.usda.gov; 2 Poultry Microbiological Safety and Processing Research Unit, Richard B. Russell Agricultural Research Center, Agricultural Research Service, U.S. Department of Agriculture, 950 College Station Road, Athens, GA 30605, USA; E-Mails: sealb@onid.oregonstate.edu (B.S.S.); johnna.garrish@ars.usda.gov (J.K.G.); boakley@westernu.edu (B.B.O.); keli.hiett@ars.usda.gov (K.H.); hungyueh.yeh@ars.usda.gov (H.-Y.Y.); eric.line@ars.usda.gov (J.E.L.); 3 Nevada Proteomics Center, University of Nevada School of Medicine, Manville Medical Building, 1664 N. Virginia Street, Reno, NV 89557-MS0330, USA; E-Mails: rebekahw@unr.edu (R.W.); schegg@unr.edu (K.M.S.)

**Keywords:** alternative antimicrobial, bacteriophage, endolysin, food safety, feed additive, peptidoglycan hydrolase, thermostabile, thermostable

## Abstract

*Clostridium perfringens* is the third leading cause of human foodborne bacterial disease and is the presumptive etiologic agent of necrotic enteritis among chickens. Treatment of poultry with antibiotics is becoming less acceptable. Endolysin enzymes are potential replacements for antibiotics. Many enzymes are added to animal feed during production and are subjected to high-heat stress during feed processing. To produce a thermostabile endolysin for treating poultry, an *E. coli* codon-optimized gene was synthesized that fused the N-acetylmuramoyl-l-alanine amidase domain from the endolysin of the thermophilic bacteriophage ΦGVE2 to the cell-wall binding domain (CWB) from the endolysin of the *C. perfringens*-specific bacteriophage ΦCP26F. The resulting protein, PlyGVE2CpCWB, lysed *C. perfringens* in liquid and solid cultures. PlyGVE2CpCWB was most active at pH 8, had peak activity at 10 mM NaCl, 40% activity at 150 mM NaCl and was still 16% active at 600 mM NaCl. The protein was able to withstand temperatures up to 50 °C and still lyse *C. perfringens*. Herein, we report the construction and characterization of a thermostable chimeric endolysin that could potentially be utilized as a feed additive to control the bacterium during poultry production.

## 1. Introduction

*Clostridium perfringens* is a Gram-positive, spore forming, anaerobic bacterium commonly present in the intestines of humans and animals. *C. perfringens* is classified into one of five types (A, B, C, D, or E) based on the toxin production. Spores of the pathogen can persist in soil, feces or the environment and the bacterium causes many severe infections of animals and humans, including food poisoning, gas gangrene, enteritis necroticans and non-foodborne gastrointestinal infections in humans. Necrotic enteritis is a peracute disease syndrome and the clinical form in poultry is caused by alpha toxin-producing *C. perfringens* type A. Some strains of *C. perfringens* type A produce an enterotoxin (CPE) during sporulation that is responsible for food-borne disease in humans [1,2,3]. In the European Union (EU) antimicrobial growth promoters (AGP) were banned from animal feeds on 1 January 2006 (Regulation 1831/2003/EC) because of concerns about the increasing prevalence of antibiotic resistances among bacteria [4,5]. Removal of these antimicrobials will dictate the need for alternative antimicrobials in order to achieve the same high level of food-animal production achieved with AGPs. Also, changes within the gastrointestinal microbial flora of food-producing animals will result in the need for a more complete understanding of the gut microbial ecology [6,7] so that appropriate antibiotic alternatives may be developed for use during food-animal production [8].

Prior to the discovery and widespread use of antibiotics, bacterial infections were treated by administering bacteriophages and were marketed by L’Oreal in France. Although Eli Lilly Co. marketed phage products for human use until the 1940s, early clinical studies with bacteriophages were not extensively explored in the United States and Western Europe after that time. Bacteriophages were, and continue to be, sold in the Russian Federation and Eastern Europe as treatments for bacterial infections [9]. With the recent surge in antibiotic resistant pathogens, there has been a resurgent interest in bacteriophage biology and use of phage or phage gene products as antibacterial agents [10,11,12,13,14] for veterinary and human medicine, as well as the bioindustry worldwide. Recently, the U.S. Food and Drug Administration approved a mixture of anti-*Listeria* viruses as a food additive to be used in processing plants for spraying onto ready-to-eat meat and poultry products to protect consumers from *Listeria monocytogenes* [15]. Although bacteriophages have been considered potentially important alternatives to antibiotics [9,16,17], it is important to emphasize that development of bacterial resistances to their viruses occurs. Evolution of phage receptors, super-infection exclusion, restriction enzyme-modification systems and abortive infection systems such as bacterial CRISPR sequences are all mechanisms that bacteriophage hosts utilize to avoid infection [18], arguing for use of bacteriophage lytic proteins.

Host strain specificity has routinely been observed relative to the bacteriophages isolated from various *C. perfringens* isolates that is probably due to evolution of the receptor and anti-receptor molecules. Consequently, several new antimicrobial agents, putative endolysins encoded by the genomes of clostridial bacteriophages have been identified in our laboratories for use as potential antimicrobials to control *C. perfringens* [14]. Two N-acetylmuramoyl-l-alanine amidases from two bacteriophages, ΦCP26F and ΦCP39O, were identical in the C-terminal cell-wall binding domain, but had only 55% identity to each other in the N-terminal catalytic domain. Both proteins, PlyCP26F and PlyCP39O, lysed their parental phage host strains of *C. perfringens* as well as other strains of the bacterium when exposed externally, but did not lyse bacteria beyond the species [19]. Recently, an endolysin from the deep-sea thermophilic bacteriophage Geobacillus virus E2 (ΦGVE2) homologous with N-acetylmuramoyl-l-alanine amidases was reported with activity over a range of temperatures from 40 to 80 °C and an optimum at 60 °C [20,21]. Herein, we report synthesis of a gene, codon optimized for *E. coli* expression that encodes the catalytic domain of the bacteriophage ΦGVE2 amidase and the cell-wall binding (CWB) domain of the endolysin encoded by the genome of ΦCP26F. Like the amidase encoded by the parental *C. perfringens* phage, the chimera protein of ΦGVE2/ΦCP26F [PlyGVE2CpCWB] lysed the bacterium in a species-specific manner.

## 2. Materials and Methods

### 2.1. Codon Optimization for the Chimeric Endolysin Synthetic Gene and Cloning Vector

**Figure 1:**
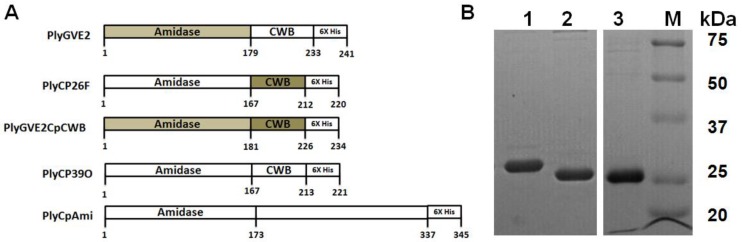
Schematic representation of the recombinant constructs and sodium dodecyl sulfate polyacrylamide gel electrophoresis (SDS-PAGE)analysis of purified proteins. (**A**) Schematic of the recombinant phage endolysins used in this report. The fusion construct PlyGVE2CpCWB consists of the amidase domain from the PlyGVE2 endolysin (light grey) and the cell-wall binding (CWB) domain of the PlyCP26F endolysin (dark grey); (**B**) Representative 15% SDS-PAGE of the endolysin constructs. Lane 1. PlyGVE2CpCWB, Lane 2. PlyCP26F, and Lane 3. PlyCP39O, M = markers (Precision Protein Plus, Biorad). All proteins are nickel column purified at >95% purity and the purified protein full length amino acid sequence was further verified by mass spectrometry (See Supplementary Information).

A fusion gene, codon optimized for expression in *E. coli*, that encoded the N-terminal 179 amino acids of PlyGVE2, the ΦGVE2 amidase endolysin [20] (NCBI accession number YP_001285830), and the C-terminal 53 amino acids from PlyCP26F (NCBI accession number YP_007004008), including a 6X His-tag [19], was synthesized by GenScript™. The gene was cloned into the pET21d expression vector (Novagen™) and used to transform BL21 (DE3) *E. coli* (Invitrogen™) by protocols previously described [19]. BLAST analyses were conducted by standard searches in NCBI [22]. The final fusion protein is presented in Figure 1 and the codon-optimized gene was submitted to GenBank with accession number 1807087.

### 2.2. Bacterial Cultures, Propagation of Strains and Protein Expression

*E. coli* strains were cultivated in LB broth utilizing standard methods and *E. coli* transformants were selected with 100 μg/mL ampicillin [23]. *C. perfringens* strains (Table 1) were typed and cultivated as previously reported [24]. The recombinant endolysin protein was expressed and purified essentially as described previously [19]. Briefly, cells from the expression host harboring the plasmid constructs were propagated in 100 mL Luria Bertani (LB) broth supplemented with 50 µg/mL ampicillin at 37 °C (shaking at 200 rpm) until the OD_600_ reading was 0.4–0.6 (log phase growth). The broth culture was held on ice for 1 h and then treated with 2 mM isopropyl-β-D-1-thiogalactopyranoside (IPTG) for induction of peptidoglycan hydrolase gene expression under control of the T7 promoter from the pET21d plasmid. The induced cells were then held overnight at 20–25 °C (slow shaking). The culture was centrifuged for 20 min at 4000 rpm. The supernatant was removed, the pellet was suspended in buffer (50 mM NaH_2_PO_4_, 300 mM NaCl, 10 mM imidazole, 10% glycerol, pH 8.0) and the suspended cells were lysed by passing through a 25 gauge needle. The resultant supernatant was purified via Nickel-NTA column chromatography [25] following manufacturer’s instructions (Qiagen™). The purified recombinant lysin and the cellular lysate were analyzed by SDS-PAGE and stained with Coomassie Blue to confirm the purity of the expressed protein [26]. Average concentrations of purified recombinant protein were ~2000 µg/mL.

**Table 1:** PlyGVE2CpCWB effectiveness against various bacteria.

Strain *^a^*	Efficacy *^b^*
*Clostridium perfringens* ATCC 12916	+
*Clostridium perfringens* ATCC 13124	+
*Clostridium perfringens* WT Cp26	+
*Clostridium perfringens* WT Cp39	+
*Clostridium sordelli* ATCC 9714	−
*Clostridium sporogenes* ATCC 3584	−
*Clostridium tetani* ATCC 19406	−
*Clostridium difficile* ATCC 43255	−
*Clostridium histolyticum* ATCC 19401	−
*Clostridium paraputrificum* ATCC 25780	−
*Clostridium septicum* ATCC 12464	−
*Listeria monocytogenes* ATCC 19114	−

*^a^* WT (wild type) strains were isolated from chicken carcass rinses; or chicken fecal material and identified by fatty acid analysis and/or; biochemical assays. ATCC, American Type Culture Collection; *^b^* Efficacy determined by plate lysis (spot) assay.

### 2.3. Identification of the Expressed Protein by LC-MS/MS and Analysis of MS Data

Approximately 20 μg of the protein was electrophoresed in a 12% Bio-Rad Mini-Protean TGX SDS electrophoresis gel and stained with Bio-Safe Coomassie (Bio-Rad) then imaged on a Bio-Rad ChemiDoc MP. Three 1.5 mm spots were cut from the protein band and excised spots were reduced and alkylated using 10 mM dithiothreitol and 100 mM iodoacetamide, then incubated with sequencing grade porcine trypsin (Promega, Fitchburg, WI, USA) in 25 mM ammonium bicarbonate overnight at 37 °C. Peptides were first separated by a Paradigm Multi-Dimensional Liquid Chromatography (MDLC) instrument (Michrom Bioresources Inc., Auburn, CA, USA), Magic C18AQ 3 μ 200Å (0.2 × 150 mm) column, (Michrom Bioresources Inc., Auburn, CA, USA) with a ZORBAX 300SB-C18 5μ (5 × 0.3 mm) trap (Agilent Technologies, Santa Clara, CA, USA). The flow rate was 2 µL/min and the solvent gradient was 5% A (5 min) to 45% B over 90 min, then 80% B (1 min). Solvent A was 0.1% aqueous formic acid and solvent B contained 0.1% formic acid in acetonitrile. Eluted peptides were analyzed using a LTQ-Orbitrap XL (Thermo Fisher Scientific, San Jose, CA, USA) equipped with a Captive Spray (Michrom Bioresources Inc.) using Xcalibur v 2.0.7. The MS was operated in data-dependent mode switching between Orbitrap-MS and LTQ-MS/MS. Full scan MS spectra (*m*/*z* 300–1800) were acquired in the positive ion mode with resolution of 60,000 in profile mode. The four most intense data-dependent peaks were subjected to MS/MS using collision-induced dissociation with a minimum signal of 50,000, isolation width of 3.0, and normalized collision energy of 35.0. Ions already selected were dynamic excluded for 30 s after a repeat count of 2 with a repeat duration of 10 s. A reject mass list was used, which included known background ions and trypsin fragments.

MS/MS samples were extracted using Sorcerer v3.5 (Sage-N Research, Milpitas, CA, USA), charge state deconvolution and deisotoping were not performed, and analyzed using Sequest (Thermo Fisher Scientific, San Jose, CA, USA version v.27, rev. 11) utilizing the predicted amino acid protein sequence. Sequest was searched with a fragment ion mass tolerance of 1.00 Da and a parent ion tolerance of 10 ppm. Iodoacetamide derivative of cysteine was specified in Sequest as a fixed modification of the C-terminus, oxidation of methionine and of the N-terminus were specified in Sequest as variable modifications. Scaffold (version Scaffold_4.0.7, Proteome Software Inc., Portland, OR, USA) validated MS/MS based peptide and protein identifications. Peptide identifications were accepted if established at greater than 95.0% probability as specified by the PeptideProphet algorithm [27]. Protein identifications were accepted if they could be established at greater than 95.0% probability and contained at least two identified peptides. Protein probabilities were assigned using the Protein Prophet algorithm [28]. The False Discovery Rate (FDR) was calculated by Scaffold using an empirical method [29].

### 2.4. Assessing Lytic Capability of the Expressed Protein

The plate lysis (spot) assay was essentially as described previously [19]. *C. perfringens* strain 12917 [30] cultures were propagated to mid-log phase (OD_600_ = 0.4–0.6) in 50 mL BHIB, where upon the cells were centrifuged at 5,000 g for 30 min. The cell pellet was washed with 50 mL lysin buffer (50 mM NH4OAc, 10 mM CaCl_2_, 1 mM DTT, pH6.2) and pelleted again. The cells were suspended in 1.0 mL lysin buffer. Ten milliliters of 50 °C semisolid BHI agar (18.5 g BHI powder, 3.5 g Bacto agar, 500 mL deionized water and autoclaved) was added to the cells and then the cells were poured into a sterile petri dish. This was allowed to sit 20 min at room temperature to solidify and then 10 μL of the Ni-chromatography purified endolysin was spotted onto the plate and allowed to air dry 20 min. The plate was incubated overnight in an anaerobic chamber at 37 °C. Additionally, plates with *C. perfringens* were allowed to incubate overnight to confluency and were then used for spot assays.

Minimal inhibitory concentration (MIC) of PlyGVE2CpCWB endolysin solution (average concentration of 2000 µg/mL) were determined by serially diluting [31,32] the endolysin in 1:2 increments from wells one through six using TMGS (10 mM Tris, pH 8, 10 mM Mg++, 0.55% NaCl, 0.1% gelatin) as a diluent in sterile Costar 3628 flat bottom, tissue culture treated, 96-well microtiter plates leaving wells with 100 µL. Untreated TMGS was used for growth control. *C. perfringens* 12917 was propagated in Brain Heart Infusion broth (BHIB) overnight [24]. The cells were pelleted at 4500 rpm for 20 min at 4 °C to remove spent culture media, then suspended in sterile BHIB to OD_600_ = 0.5. A 1:15 dilution of the adjusted cell suspension was then made in sterile BHIB resulting in a final inoculum between 10^5^ and 10^6^ cfu/mL. Subsequently, 10 µL was then dispensed into all treated and positive control wells of the microtiter plate yielding total volumes of 110 µL. Finally, at least one well was left untreated and un-inoculated for negative control. The microtiter plate was incubated under anaerobic condition for 24 h at 37 °C. Following incubation, wells were visually inspected using a mirrored stand under white light. The MIC was recorded as the lowest enzyme concentration showing no growth (clear well by eye) of *C. perfringens*. To observe any morphological changes following treatment, a 900 µL aliquot of *C. perfringens* 12917 at 10^6^ cells per mL was incubated with PlyGVE2CpCWB 100 µL (conc. 2000 µg/mL) for 30 min and 10 µL was spotted on a slide for Gram staining of cells.

A modified turbidity reduction assay [33] was completed using *C. perfringens* 12917 propagated anaerobically to mid-log phase (OD_600_ = 0.4–0.6) in BHIB media at 37 °C. The cells were pelleted (3000 rpm, 15 min, 4 °C), washed three times in sterile distilled water or buffer, and then suspended to an OD_600_ of ~2. For pH assays, bacterial cells were washed and suspended in sterile distilled water, and the endolysin was prepared in aliquots of 40 mM boric acid-phosphoric acid buffer (BP) covering the pH range tested, pH 4 to pH 11. For NaCl studies, the cells were prepared in 50 mM NaH_2_PO_4_, pH 8.0, and the endolysin was prepared in 50 mM NaH_2_PO_4_ 2× NaCl, pH 8. For thermostability assays, the cells were prepared in 50 mM NaH_2_PO_4_, pH 8, and the endolysin was prepared in 50 mM NaH_2_PO_4_, 20 mM NaCl, pH 8, then mixed for a final concentration of 10 mM NaCl. In the wells of a 96 well plate, 0.1 mL cells were added to 0.1 mL of 0.2 mg/mL endolysin and lytic activity was determined by a decrease in the absorbance at OD_600_ of the cell suspension in a SpectraMax 340 plate reader (Molecular Devices, Sunnyvale, CA, USA) for 20 min at 22 °C, taking readings every 20 s. Activity was calculated from the Vmax determined from the linear portion of each lysis curve using the Softmax Pro software (Molecular Devices, Sunnyvale, CA, USA), and data was normalized to the maximal activity from each experiment [34]. Data points were obtained from triplicate data points in each of three experiments. For thermostability studies, the enzyme (200 µg/mL PlyGVE2CpCWB or 40 µg/mL PlyCP26F) was incubated for 30 min at the target temperature in lysin buffer, placed on ice 10 min, before being assayed at 22 °C for lytic activity against *C. perfringens*.

## 3. Results and Discussion

### 3.1. Expression, Purification, and Confirmation of PlyGVE2CpCWB

BLAST analyses of both PlyGVE2 and PlyCP26F endolysins predicted that the proteins were N-acetylmuramoyl-l-alanine amidases or MurNAc-LAA (also known as peptidoglycan aminohydrolase, NAMLA amidase, NAMLAA, Amidase 3, and peptidoglycan amidase; EC 3.5.1.28). Those enzymes where the MurNAc-LAA domain has been analyzed, have been shown to hydrolyze the amide bond between N-acetylmuramoyl moiety and the first l-amino acid (usually alanine) in the Gram positive bacterial cell wall peptidoglycan [35]. Members of this class of endolysins have no signal peptides and their translocation through the cytoplasmic membrane is thought to proceed with the help of phage-encoded holin proteins. The amidase catalytic module was fused to another modular domain, a CWB, at the C-terminus, which is responsible for high affinity binding of the protein to the cell wall [11,36]. Both of the bacteriophage endolysins reported herein had the E residue at position 89 of the PlyGVE2 and at position 87 for the clostridial bacteriophage endolysins that has been determined necessary for amidase activity [37]. Interestingly the C-terminal CWB of the clostridial bacteriophage endolysins was predicted by BLAST analyses to be homologous to the RNA recognition motif (RRM) superfamily of eukaryotic proteins involved in post-transcriptional gene expression processes [38]. However, what role, if any, this motif plays in bacterial interactions is unknown. Hereafter, we will refer to the ΦGVE2 endolysin as PlyGVE2, the ΦCP26F endolysin as PlyCP26F, and the ΦCP39O endolysin as PlyCP39O.

A codon optimized gene for the PlyGVE2 predicted N-acetylmuramoyl-l-alanine amidase endolysin domain (179 amino acids) [20] was synthesized in-frame with the CWB domain (53 amino acids) of PlyCP26F from the *Clostridium perfringens*-specific bacteriophage ΦCP26F (identical to the PlyCP39O endolysin CWB domain) [19]. The expressed protein designated PlyGVE2CpCWB was 242 amino acids in length including the LE-6X His-tag C-terminus for purification using Ni-chromatography (Figure 1A). The expressed protein had a predicted molecular weight 27,261 Daltons, which corresponded to the size as determined by SDS-PAGE (Figure 1B) that was confirmed by proteomics analyses of the amino acid sequence (Supplementary information). The recombinant PlyGVE2CpCWB was purified by Ni-affinity chromatography (Figure 1B) and concentrations of the protein produced in *E. coli* varied from 1700 to 3000 µg/mL with routine concentrations of 2000 µg/mL achieved.

### 3.2. Lysis of *C. Perfringens* by PlyGVE2CpCWB

**Figure 2:**
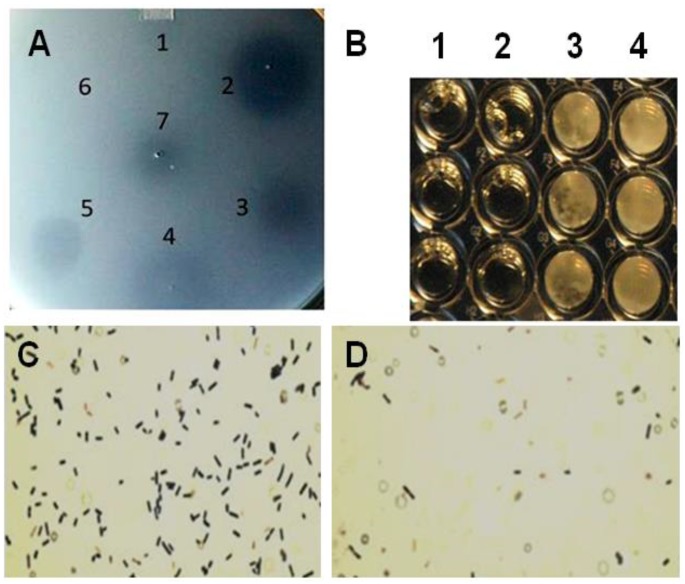
Plate lysis, minimal inhibitory concentration and Gram-stain of lytic enzyme treated *Clostridium perfringens* 12917. (**A**) Plate (spot) lysis assay 10 µL spots: 1. PlyCpAmi (8 μg); 2. PlyGVE2CpCWB (10 μg) and lysozyme (2.5 μg); 3. PlyGVE2CpCWB (20 μg); 4. Lysozyme (5 μg); 5. PlyCP39O (5 μg); 6. Ampicillin (5 μg); and 7. Lower concentration PlyGVE2CpCWB (4 μg); (**B**) Minimal inhibitory concentration (MIC) for the recombinant PlyGVE2CpCWB protein. A representative MIC assay is illustrated that was determined by serially diluting the endolysin in 1:2 increments using diluent in sterile flat bottom, tissue culture treated, 96-well microtiter plates leaving wells with 100 µL. Well concentrations: 1. 2500 µg/mL, 2. 1250 µg/mL, 3. 625 µg/mL, and 4. 312.5 µg/mL. Buffer alone was used for control; (**C**) Gram-stain image of *C. perfringens* 12917 following treatment with PlyGVE2CpCWB protein. The bacterium was untreated (Panel C) or treated (Panel D) with the purified recombinant protein. Gram stain magnification is 1000×, as the 100× oil objective was used with a 10× eyepiece magnification.

The recombinant protein was utilized in a plate-spot assay to determine if the expressed protein was capable of lysing the bacterium *C. perfringens* (Figure 2A). The spot assays were completed utilizing confluent lawns of *C. perfringens* that were propagated overnight under anaerobic conditions at 37 °C. The spot assays were completed with other recombinant proteins reported to lyse the bacterium such as the PlyCpAmi [39] along with the PlyGVE2CpCWB and lysozyme or the PlyGVE2CpCWB alone and lysozyme alone. Lysozyme has been previously reported to act on *C. perfringens* [40], and sometimes combinations of enzymes show greater lysis [41]. Our previously reported bacteriophage endolysin PlyCP39O [19] and ampicillin were also utilized for the spot assay (Figure 2A). All the antimicrobial agents including lysozyme produced clear spots on confluent *C. perfringens* cells with the exception of ampicillin that requires actively dividing cells for its antimicrobial activity [42].

MICs were determined by serial 1:2 dilution of the PlyGVE2CpCWB (2000 µg/mL) with approximately 10^6^ *C. perfringens*, target concentrations ranged from 5.55 × 10^5^ to 9.0 × 10^6^, followed by incubation for 24 h at 37 °C. MICs were replicated in triplicate in three separate assays resulting in a range of 1000 ± 250 µg/mL (Figure 2B). The MIC values are greater than traditional antibiotics for *C. perfringens* isolates recovered from poultry [43,44]. Lysozyme was capable of lysing *C. perfringens* in the spot assay, and in combination with PlyGVE2CpCWB, it appeared to create a larger zone of clearing (Figure 2A, spot 2 *vs.* spots 3 and 4). The spot assay is less quantitative than the MIC assay, so next we tried this in a MIC assay. When lysozyme was used in combination with PlyGVE2CpCWB, the results were inconclusive due to a precipitation/aggregation effect (data not shown). This was unfortunate for two reasons: first, the lysozyme MIC reported by Zhang *et al.* [40] was 156 µg/mL, and by adding this compound to the MIC assays we hoped to see improvement in the MIC results. Second, *in vivo*, supplementation of chicken feed with 40 mg lysozyme/kg for animals challenged with *C. perfringens* reportedly reduced the concentration of the bacterium in the ileum, reduced the intestinal lesion scores and improved feed conversion [45]. Since lysozyme has been proven useful in chicken feed, it might be desirable to combine it with PlyGVE2CpCWB for this application.

The Gram stain of endolysin treated and untreated cells was completed to illustrate the cell lysis effect achieved by exposing *C. perfringens* cells to the recombinant PlyGVE2CpCWB (Figure 2C,D). As reported previously for other *C. perfringens* bacteriophage endolysins [19,39,46,47,48], PlyGVE2CpCWB treated *C. perfringens* cells were completely digested, and non-intact compared to the untreated cells.

The specificity of PlyGVE2CpCWB was tested against different species of bacteria and different strains of *C. perfringens*. PlyGVE2CpCWB was effective against four strains of *C. perfringens*, two of which were isolates from chicken carcasses or fecal matter (Table 1), indicating the potential for *in vivo* use against *C. perfringens* in poultry. Seven other species of *Clostridium* and the bacterium *Listeria monocytogenes* were not lysed by PlyGVE2CpCWB. This is the same specificity reported for PlyCP26F, the source of the CBD present in PlyGVE2CpCWB, suggesting that the CWB confers specie-specificity on the fusion construct, (as has been reported for other heterologous phage endolysins [35]) although the ability of the parental PlyGVE2 endolysin to lyse *C. perfringens* was not tested.

### 3.3. Characterization of PlyGVE2CpCWB Activity

**Figure 3:**
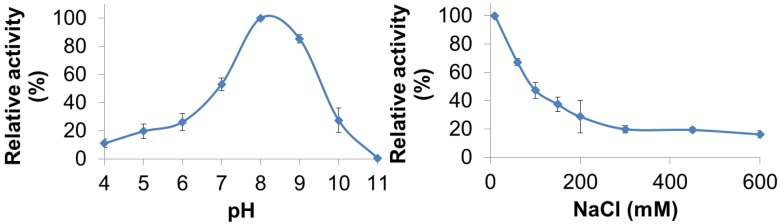
Effect of pH and NaCl on the ability of PlyGVE2CpCWB to lyse *Clostridium perfringens*. Top panel shows the activity of the endolysin over a range of pH values. Bottom panel shows the activity of the endolysin over a range of NaCl concentrations. Lytic activity of the endolysin was determined by the turbidity reduction assay and all activities were normalized to the maximal activity achieved (=100%).

PlyGVE2CpCWB lytic activity was characterized for pH and NaCl concentration dependence by utilizing turbidity reduction assays. The pH range for PlyGVE2CpCWB was assayed from pH 4.0 to 11.0 and substantial activity (10%–80% of maximum) was observed from pH 4.0 to pH 10.0 with the greatest activity occurring at pH 8.0 (Figure 3, top panel). Interestingly, the PlyGVE2 endolysin, from which the catalytic domain of PlyGVE2CpCWB is derived, had a broad pH range for activity, but had greatest activity at pH 6.0 [20]. Some differences were expected between PlyGVE2 and PlyGVE2CpCWB because of the substitution of the CWB domain and because the PlyGVE2 endolysin was characterized at 60 °C. Since PlyGVE2CpCWB maintains activity at low pH, it may well survive passage through the gizzard of a chicken, pH ~3, into the intestines, which are between pH 6.0 and 6.8 [49] and once there, it could then be able to lyse *C. perfringens* that might be present in the gastrointestinal tract. Based on the pH data, further characterization, for NaCl concentration dependence and thermostability, was completed at pH 8.0. Since NaCl in solution can have an effect on enzyme solubility and activity, we next examined the influence of NaCl on the activity of PlyGVE2CpCWB over a range of 10 mM to 600 mM NaCl in 50 mM NaH_2_PO_4_ pH 8.0 (Figure 3, bottom panel). Interestingly, the highest lytic activity was observed at the lowest salt concentration, 10 mM NaCl. Lytic activity decreased with the increase of salt in solution, with activity decreasing to 37% maximal at 150 mM NaCl, and still retained 16% activity at 600 mM NaCl. *Clostridium perfringens* has been reported in the caeca of broiler chickens [50]. The caeca, a pair of sacs or extensions off of the chicken intestine, have been reported to have 27–64 mM sodium ions, 22–36 mM potassium ions, and 17–25 mM chloride ions, depending on the diet of the chickens [51]. This low concentration of sodium chloride would be compatible with PlyGVE2CpCWB activity in the caeca.

**Figure 4:**
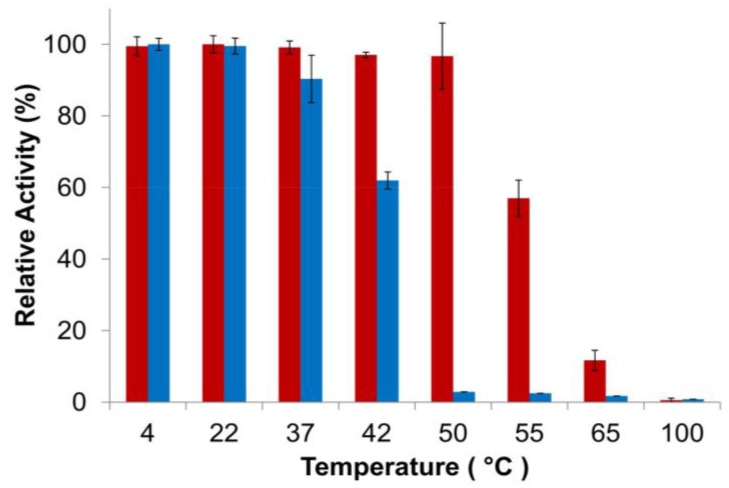
Thermostability of PlyGVE2CpCWB and PlyCP26F. The proteins were incubated at various temperatures for 30 min, placed on ice for 10 min, and activity was then determined by the standard turbidity reduction assay against *C. perfringens* normalized to the maximal activity achieved (=100%). Red bars are PlyGVE2CpCWB. Blue bars are PlyCP26F.

Finally, thermostability of the PlyGVE2CpCWB fusion *vs.* the parental ΦCP26F endolysin was examined using the turbidity reduction assay. It is not practical to use *Clostridium perfringens* cells as substrate to test for activity at high temperatures. Instead the endolysins were incubated at various temperatures and then allowed to cool down before performing the standard assay (which is more in keeping with the scenario if these enzymes were to be used as a feed additive). In this context, thermostability refers to either the maintaining of protein structure at high temperature or the ability to refold after incubation at high temperature or regaining activity when cooled down. Add enough heat to a protein, and it will eventually unfold. The unfolding of proteins and/or the failure to refold can lead to the formation of aggregates of inactive protein. Endolysin concentrations were chosen to give similar levels of activity during the turbidity reduction assay. Heat treatment of the endolysins occurred at 2× assay concentrations, 200 μg/mL for PlyGVE2CpCWB and 40 μg/mL for PlyCP26F. Higher protein concentrations tend to favor formation of aggregates [52]. This concentration dependent response to heat treatment was reported for a staphylokinase variant which had substantial loss of activity after incubation at 60 °C for one hour at 0.5 mg/mL, but had no loss of activity at a concentration of 0.08 mg/mL. In another study, lysozyme was shown to lose activity at 90 °C more rapidly when the concentration of lysozyme was increased [53]. Since PlyGVE2CpCWB was heat treated at higher concentrations than was PlyCP26F, it should have been more prone to aggregate formation, and therefore display a lower tolerance to heat treatment. However, the data show that PlyGVE2CpCWB is substantially more tolerant to heat treatment than PlyCP26F (Figure 4). It was reported that the parental endolysin PlyGVE2, from the thermophilic bacteriophage ΦGVE2, retained ~80% activity against its host strain after 30 min incubation at 55 °C [20]. By comparison, the other parental enzyme, PlyCP26F, was completely inactivated after 30 min at 55 °C and more than 40% inactivated at 50 °C. Similarly, PlyC, a potent endolysin lytic for *Streptococcus pyogenes*, is completely inactive after 30 min at 50 °C [54]. PlyGVE2CpCWB maintained greater than 95% activity after a 30 min incubation at 50 °C, and retained 57% activity after a 55 °C incubation (Figure 4), suggesting that the PlyCP26F CWB domain reduces the thermostability of the fusion protein compared to the parental PlyGVE2 endolysin, but improves the thermostability compared to the parental PlyCP26F endolysin. This improvement in thermostability is useful in the context of treating chickens with gastrointestinal tract infections. Chickens have a rectal temperature between 40.6 and 43.0 °C [55,56], and this likely represents the temperature in their intestines. PlyCP26F loses roughly 40% of its activity after 30 min at 42 °C, while PlyGVE2CpCWB is still fully active. This indicates that PlyGVE2CpCWB is better suited to survive the temperature inside the chicken, allowing it more time to kill any *Clostridium perfringens* that are present.

## 4. Conclusions

A recombinant protein designated PlyGVE2CpCWB was expressed from a synthesized gene (codon optimized for expression in *E. coli*) that included the N-terminus (predicted enzymatic domain) of a thermophilic bacteriophage ΦGVE2 endolysin (amidase) and the conserved cell-wall binding domain of a *Clostridium perfringens*-specific bacteriophage ΦCP26F endolysin. The recombinant protein lysed the bacterium *C. perfringens* in both plate and liquid lysis assays. PlyGVE2CpCWB had the same target cell specificity as PlyCP26F, the source of its cell-wall binding domain. PlyGVE2CpCWB had activity at a pH from 6 to 10 with peak activity at pH 8.0, and was active over a wide range of saline conditions. PlyGVE2CpCWB was substantially more resistant to elevated temperatures than was PlyCP26F. This is the first example of an endolysin from a thermophilic bacteriophage that was re-targeted to a poultry gut pathogen with obvious implications for its potential use in animal feed.

## Supplementary Files

Supplementary File 1

## References

[1] Smedley J.G., Fisher D.J., Sayeed S., Chakrabarti G., McClane B.A. (2004). The enteric toxins of *Clostridium perfringens*. Rev. Physiol. Biochem. Pharmacol..

[2] Sawires Y.S., Songer J.G. (2006). *Clostridium perfringens*: Insight into virulence evolution and population structure. Anaerobe.

[3] Scallan E., Griffin P.M., Angulo F.J., Tauxe R.V., Hoekstra R.M. (2011). Foodborne illness acquired in the United States—Unspecified agents. Emerg. Infect. Dis..

[4] Huyghebaert G., Ducatelle R., van Immerseel F. (2011). An update on alternatives to antimicrobial growth promoters for broilers. Vet. J..

[5] Millet S., Maertens L. (2011). The European ban on antibiotic growth promoters in animal feed: From challenges to opportunities. Vet. J..

[6] Wise M.G., Siragusa G.R. (2007). Quantitative analysis of the intestinal bacterial community in one- to three-week-old commercially reared broiler chickens fed conventional or antibiotic-free vegetable-based diets. J. Appl. Microbiol..

[7] Oakley B.B., Morales C.A., Line J., Berrang M.E., Meinersmann R.J., Tillman G.E., Wise M.G., Siragusa G.R., Hiett K.L., Seal B.S. (2013). The poultry-associated microbiome: Network analysis and farm-to-fork characterizations. PLoS ONE.

[8] Seal B.S., Lillehoj H.S., Donovan D.M., Gay C.G. (2013). Alternatives to antibiotics: A symposium on the challenges and solutions for animal production. Anim. Health Res. Rev..

[9] Sulakvelidze A. (2005). Phage therapy: An attractive option for dealing with antibiotic-resistant bacterial infections. Drug Discov. Today.

[10] Liu J., Dehbi M., Moeck G., Arhin F., Bauda P., Bergeron D., Callejo M., Ferretti V., Ha N., Kwan T. (2004). Antimicrobial drug discovery through bacteriophage genomics. Nat. Biotechnol..

[11] Pastagia M., Schuch R., Fischetti V.A., Huang D.B. (2013). Lysins: The arrival of pathogen-directed anti-infectives. J. Med. Microbiol..

[12] Schmelcher M., Donovan D.M., Loessner M.J. (2012). Bacteriophage endolysins as novel antimicrobials. Future Microbiol..

[13] Rodríguez-Rubio L., Martínez B., Donovan D.M., Rodríguez A., García P. (2013). Bacteriophage virion-associated peptidoglycan hydrolases: Potential new enzybiotics. Crit. Rev. Microbiol..

[14] Seal B.S. (2013). Characterization of bacteriophages virulent for *Clostridium perfringens* and identification of phage lytic enzymes as alternatives to antibiotics for potential control of the bacterium. Poult. Sci..

[15] Bren L. (2007). Bacteria-eating virus approved as food additive. FDA Consum..

[16] Lu T.K., Koeris M.S. (2011). The next generation of bacteriophage therapy. Curr. Opin. Microbiol..

[17] Maura D., Debarbieux L. (2011). Bacteriophages as twenty-first century antibacterial tools for food and medicine. Appl. Microbiol. Biotechnol..

[18] Labrie S.J., Samson J.E., Moineau S. (2010). Bacteriophage resistance mechanisms. Nat. Rev. Microbiol..

[19] Simmons M., Donovan D.M., Siragusa G.R., Seal B.S. (2010). Recombinant expression of two bacteriophage proteins that lyse *Clostridium perfringens* and share identical sequences in the C-terminal cell wall binding domain of the molecules but are dissimilar in their N-terminal active domains. J. Agric. Food Chem..

[20] Ye T., Zhang X. (2008). Characterization of a lysin from deep-sea thermophilic bacteriophage GVE2. Appl. Microbiol. Biotechnol..

[21] Jin M., Ye T., Zhang X. (2013). Roles of bacteriophage GVE2 endolysin in host lysis at high temperatures. Microbiology.

[22] Schäffer A.A., Aravind L., Madden T.L., Shavirin S., Spouge J.L., Wolf Y.I., Koonin E.V., Altschul S.F. (2001). Improving the accuracy of PSI-BLAST protein database searches with composition-based statistics and other refinements. Nucleic Acids Res..

[23] Studier F.W., Moffatt B.A. (1986). Use of bacteriophage T7 RNA polymerase to direct selective high-level expression of cloned genes. J. Mol. Biol..

[24] Siragusa G.R., Danyluk M.D., Hiett K.L., Wise M.G., Craven S.E. (2006). Molecular subtyping of poultry-associated type A *Clostridium perfringens* isolates by repetitive-element PCR. J. Clin. Microbiol..

[25] Crowe J., Döbeli H., Gentz R., Hochuli E., Stüber D., Henco K. (1994). 6xHis-Ni-NTA chromatography as a superior technique in recombinant protein expression/purification. Methods Mol. Biol..

[26] Hames B.D., Hames B.D., Rickwood D. (1990). One-dimensional polyacrylamide gel electrophoresis. Gel Electrophoresis of Proteins: A Practical Approach.

[27] Keller A., Nesvizhskii A.I., Kolker E., Aebersold R. (2002). Empirical statistical model to estimate the accuracy of peptide identifications made by MS/MS and database search. Anal. Chem..

[28] Nesvizhskii A.I., Keller A., Kolker E., Aebersold R. (2003). A statistical model for identifying proteins by tandem mass spectrometry. Anal. Chem..

[29] Kall L., Storey J.D., MacCoss M.J., Noble W.S. (2008). Assigning significance to peptides identified by tandem mass spectrometry using decoy databases. J. Proteome Res..

[30] Hobbs B.C., Smith M.E., Oakley C.L., Warrack G.H., Cruickshank J.C. (1953). *Clostridium welchii* food poisoning. J. Hyg..

[31] Rotilie C.A., Fass R.J., Prior R.B., Perkins R.L. (1975). Microdilution technique for antimicrobial susceptibility testing of anaerobic bacteria. Antimicrob. Agents Chemother..

[32] Andrews J.M. (2001). Determination of minimum inhibitory concentrations. J. Antimicrob. Chemother..

[33] Donovan D.M., Dong S., Garrett W., Rousseau G.M., Moineau S., Pritchard D.G. (2006). Peptidoglycan hydrolase fusions maintain their parental specificities. Appl. Environ. Microbiol..

[34] Linden S.B., Zhang H., Heselpoth R.D., Shen Y., Schmelcher M., Eichenseher F., Nelson D.C. (2015). Biochemical and biophysical characterization of PlyGRCS, a bacteriophage endolysin active against methicillin-resistant *Staphylococcus aureus*. Appl. Microbiol. Biotechnol..

[35] Becker S.C., Dong S., Baker J.R., Foster-Frey J., Pritchard D.G., Donovan D.M. (2009). LysK CHAP endopeptidase domain is required for lysis of live staphylococcal cells. FEMS Microbiol. Lett..

[36] Hermoso J.A., García J.L., García P. (2007). Taking aim on bacterial pathogens: From phage therapy to enzybiotics. Curr. Opin. Microbiol..

[37] Low L.Y., Yang C., Perego M., Osterman A., Liddington R.C. (2005). Structure and lytic activity of a Bacillus anthracis prophage endolysin. J. Biol. Chem..

[38] Bousquet-Antonelli C., Deragon J.M. (2009). A comprehensive analysis of the La-motif protein superfamily. RNA.

[39] Tillman G.E., Simmons M., Garrish J.K., Seal B.S. (2013). Expression of a *Clostridium perfringens* genome-encoded putative N-acetylmuramoyl-l-alanine amidase as a potential antimicrobial to control the bacterium. Arch. Microbiol..

[40] Zhang G., Darius S., Smith S.R., Ritchie S.J. (2006). *In vitro* inhibitory effect of hen egg white lysozyme on *Clostridium perfringens* type A associated with broiler necrotic enteritis and its alpha-toxin production. Lett. Appl. Microbiol..

[41] Schmelcher M., Powell A.M., Becker S.C., Camp M.J., Donovan D.M. (2012). Chimeric phage lysins act synergistically with lysostaphin to kill mastitis-causing *Staphylococcus aureus* in murine mammary glands. Appl. Environ. Microbiol..

[42] Waxman D.J., Stromingerr J.L. (1983). Penicillin-binding proteins and the mechanism of action of beta-lactam antibiotics. Annu. Rev. Biochem..

[43] Watkins K.L., Shryock T.R., Dearth R.N., Saif Y.M. (1997). *In-vitro* antimicrobial susceptibility of *Clostridium perfringens* from commercial turkey and broiler chicken origin. Vet. Microbiol..

[44] Slavić D., Boerlin P., Fabri M., Klotins K.C., Zoethout J.K., Weir P.E., Bateman D. (2011). Antimicrobial susceptibility of *Clostridium perfringens* isolates of bovine, chicken, porcine, and turkey origin from Ontario. Can. J. Vet. Res..

[45] Liu D., Guo Y., Wang Z., Yuan J. (2010). Exogenous lysozyme influences *Clostridium perfringens* colonization and intestinal barrier function in broiler chickens. Avian Pathol..

[46] Gervasi T., Horn N., Wegmann U., Dugo G., Narbad A., Mayer M.J. (2014). Expression and delivery of an endolysin to combat *Clostridium perfringens*. Appl. Microbiol. Biotechnol..

[47] Schmitz J.E., Ossiprandi M.C., Rumah K.R., Fischetti V.A. (2011). Lytic enzyme discovery through multigenomic sequence analysis in *Clostridium perfringens*. Appl. Microbiol. Biotechnol..

[48] Zimmer M., Vukov N., Scherer S., Loessner M.J. (2002). The murein hydrolase of the bacteriophage phi3626 dual lysis system is active against all tested *Clostridium perfringens* strains. Appl. Environ. Microbiol..

[49] Kokas E., Phillips J.L., Brunson W.D. (1967). The secretory activity of the duodenum in chickens. Comp. Biochem. Physiol..

[50] Lu J., Idris U., Harmon B., Hofacre C., Maurer J.J., Lee M.D. (2003). Diversity and succession of the intestinal bacterial community of the maturing broiler chicken. Appl. Environ. Microbiol..

[51] Rolls B.A. (1977). Inorganic ions in the intestinal and caecal contents of germ-free and conventional chickens. Lab. Anim..

[52] Zettlmeissl G., Rudolph R., Jaenicke R. (1979). Reconstitution of lactic dehydrogenase. Noncovalent aggregation *vs.* reactivation. 1. Physical properties and kinetics of aggregation. Biochemistry.

[53] Nohara D., Mizutani A., Sakai T. (1999). Kinetic study on thermal denaturation of hen egg-white lysozyme involving precipitation. J. Biosci. Bioeng..

[54] Heselpoth R.D., Nelson D.C. (2012). A new screening method for the directed evolution of thermostable bacteriolytic enzymes. J. Vis. Exp..

[55] Normal Rectal Temperature Ranges Reference Guides Merck Vet Manual. http://www.merckvetmanual.com/mvm/appendixes/reference_guides/normal_rectal_temperature_ranges.html.

[56] Robertshaw D., Reece W.O. (2004). Temperature Regulation and Thermal Environment. Dukes’ Physiology of Domestic Animals.

