# Supplementary material for: A Thermophilic Phage Endolysin Fusion to a Clostridium perfringens-Specific Cell Wall Binding Domain Creates an Anti-Clostridium Antimicrobial with Improved Thermostability
Source: Viruses. 2015 Jun 12;7(6):3019–34. doi: 10.3390/v7062758 (PMC4488725; doi:10.3390/v7062758)
Supplement: Supplementary File 1 [file viruses-07-02758-s001.pdf]

Supplementary Data for "A thermophilic phage endolysin fusion to a Clostridium perfringens-specific cell wall binding domain creates an anti-clostridium antimicrobial with improved thermostability"

Recombinant_protein_20130729: GAV-CpCWB Mass Spectrometry and BLAST Results

Original Sequence:

MGMKKIFWDKGHGGSDPGAVANGLQEKNLTHKIVEYATDYLAAHYEGFTQRVSREGDQSLTLDQRADMANKWGADVFVSVHINAGK
GTGFEIYVHPNASPQSIALQNVLHGEILSAMRQFGNITDRGKKRANYAVLRETKMPAVLTENLFIDSNDAKHLKNEAFLKAVGEAHARGVA
KFLGRYLANAIDPNIPLEKEQDYYRVCVQRFTNKEDAEKAQQRISNELGYYCFAEKI

putative N-acetylmuramoyl-L-alanine amidase [Geobacillus virus E2]
Sequence ID: ref|YP_001285830.1| Length: 233; Number of Matches: 1
Gene-associated gene details
Range 1: 1 to 230 GenPeptGraphics

Alignment statistics for match #1

Score	Expect	Method	Identities	Positives	Gaps
386 bits(991)	4e-133	Compositional matrix adjust.	193/231(84%)	202/231(87%)	4/231(1%)

```
Query 3     MKKIFWDKGHGGSDPGAVANGLQEKNLTHKIVEYATDYLAAHYEGFTQRVSREGDQSLTL   62
            MKKIFWDKGHGGSDPGAVANGLQEKNLTHKIVEYATDYLAAHYEGFTQRVSREGDQSLTL
Sbjct 1     MKKIFWDKGHGGSDPGAVANGLQEKNLTHKIVEYATDYLAAHYEGFTQRVSREGDQSLTL   60

Query 63    DQRADMANKWGADVFVSVHINAGKGTGFEIYVHPNASPQSIALQNVLHGEILSAMRQFGN  122
            DQRADMANKWGADVFVSVHINAGKGTGFEIYVHPNASPQSIALQNVLHGEILSAMRQFGN
Sbjct 61    DQRADMANKWGADVFVSVHINAGKGTGFEIYVHPNASPQSIALQNVLHGEILSAMRQFGN  120

Query 123   ITDRGKKRANYAVLRETKMPAVLTENLFIDSNDAKHLKNEAFLKAVGEAHARGVAKFLGR  182
            ITDRGKKRANYAVLRETKMPAVLTENLFIDSNDAKHLKNEAFLKAVGEAHARGVAKFLG
Sbjct 121   ITDRGKKRANYAVLRETKMPAVLTENLFIDSNDAKHLKNEAFLKAVGEAHARGVAKFLGL  180

Query 183   YLANAIDPNI-PLEKEQD--YYRVCVQRFTNKEDAEKAQQRISNELGYYCFAEKI      232
                   P   P +K  D   YRV V   F ++E+AE+   +  + GY   +
Sbjct 181   KEKQKAQPEAKPQQKPSDKKLYRVQVGAFADRENAERLAEELKRK-GYPVY---ITD     233
```

Mass Spec Sequences:

mgmkkIFWDKGHGGSDPGAVANGLQEKNLTHKIVEYATDYLAAHYEGFTQRvsrEGDQSLTLDQRadmankwgadvfvsvhinagkGTGFEIY
VHPNASPQSIALQNVLHGEILSAMRqfgnitdrgkkranyavlretkMPAVLTENLFIDSNDAKhlkNEAFLKAVGEAHARgvakflgrYLANAIDPNI
PLEKEQDYYRvcvqrftnkedaekaqqrISNELGYYCFAEKilehhhhhh

Underlined sequence is Geobacillus phage E2 Amidase, while remaining C-terminal sequences are the cell wall binding domain for *Clostridium perfringens*; Sequences in CAPS indicate peptides identified by mass spec.

DATABASE SEARCHING-- Tandem mass spectra were extracted by Sorcerer v3.5 (Sage-N Research, Milpitas CA). Charge state deconvolution and deisotoping were not performed. All MS/MS samples were analyzed using Sequest (Thermo Fisher Scientific, San Jose, CA, USA; version 1.0). Sequest was set up to search the Clostridium_Seal_decoys_20130729 database assuming the digestion enzyme strict-trypsin. Sequest was searched with a fragment ion mass tolerance of 1.00 Da and a parent ion tolerance of 10.0 PPM. Carbamidomethyl of cysteine was specified in Sequest as a fixed modification. Oxidation of methionine was specified in Sequest as a variable modification.

CRITERIA FOR PROTEIN IDENTIFICATION-- Scaffold (version Scaffold_4.0.7, Proteome Software Inc., Portland, OR) was used to validate MS/MS based peptide and protein identifications. Peptide identifications were accepted if they could be established at greater than 95.0% probability by the Scaffold Local FDR algorithm. Protein identifications were accepted if they could be established at greater than 95.0% probability and contained at least 5 identified peptides. Protein probabilities were assigned by the Protein Prophet algorithm (Nesvizhskii, AI et al Anal. Chem. 2003;75(17):4646-58). Proteins that contained similar peptides and could not be

differentiated based on MS/MS analysis alone were grouped to satisfy the principles of parsimony. Proteins sharing significant peptide evidence were grouped into clusters. Proteins were annotated with GO terms from NCBI (downloaded Jul 30, 2013). (Ashburner, M et al Nat. Genet. 2000;25(1):25-9).

3369-Seal_20percent_Condensed Displaying: Protein Identification Probability

Identified Proteins (1)		Accession Number		Molecular Weight	Protein Grouping Ambiguity	Quantitative Variance	Taxonomy	Bio-Sample 1
1	true	Empty	recombinant_protein_20130729	GAV-CpCWB	27 kDa			100%

Index	Peptide	Prob	Exclusive To	Valid	GAV-CpCWB
01	ANYAVLR	67%	recombinant_protein_20130729	true	1.00
02	ANYAVLRETK	13%	recombinant_protein_20130729	true	1.00
03	AVGEAHAR	99%	recombinant_protein_20130729	true	1.00
04	EGDQSLTLDQR	100%	recombinant_protein_20130729	true	1.00
05	EQDYYR	48%	recombinant_protein_20130729	true	1.00
06	FLGRYLANAIDPNIPLEK	14%	recombinant_protein_20130729	true	1.00
07	FTNKEDAEK	14%	recombinant_protein_20130729	true	1.00
08	GHGGSDPGAVANGLQEK	100%	recombinant_protein_20130729	true	1.00
09	GHGGSDPGAVANGLQEKNLTHK	72%	recombinant_protein_20130729	true	1.00
10	GTGFEIYVHPNASPQSIALQNVLHGEILSAMR	100%	recombinant_protein_20130729	true	1.00
11	GVAKFLGR	11%	recombinant_protein_20130729	true	1.00
12	HLKNEAFLK	76%	recombinant_protein_20130729	true	1.00
13	IFWDK	10%		true	
14	IFWDKGHGGSDPGAVANGLQEK	100%	recombinant_protein_20130729	true	1.00
15	ISNELGYYCFAEK	100%	recombinant_protein_20130729	true	1.00
16	IVEYATDYLAAHYEGFTQR	100%	recombinant_protein_20130729	true	1.00
17	IVEYATDYLAAHYEGFTQRVSR	13%	recombinant_protein_20130729	true	1.00
18	KIFWDK	13%	recombinant_protein_20130729	true	1.00
19	MPAVLTENLFIDSNDAK	100%	recombinant_protein_20130729	true	1.00
20	NEAFLKAVGEAHAR	100%	recombinant_protein_20130729	true	1.00
21	NLTHKIVEYATDYLAAHYEGFTQR	100%	recombinant_protein_20130729	true	1.00
22	NYAVLR	31%	recombinant_protein_20130729	true	1.00
23	QFGNITDR	13%	recombinant_protein_20130729	true	1.00
24	RANYAVLR	82%	recombinant_protein_20130729	true	1.00
25	WGADVFVSVHINAGK	52%	recombinant_protein_20130729	true	1.00
26	YLANAIDPNIPLEK	100%	recombinant_protein_20130729	true	1.00
27	YLANAIDPNIPLEKEQDYYR	100%	recombinant_protein_20130729	true	1.00
28	YLANAIDPNIPLEKEQDYYRVCVQR	12%	recombinant_protein_20130729	true	1.00

Experiment name	Biological sample category		Biological sample name	MS/MS sample name	Protein group	Protein accession number		Protein name	Protein identification probability		Protein percentage of total spectra		Protein molecular weight (AMU)	
	Number of unique peptides		Number of unique spectra		Number of total spectra		Spectrum name	Peptide sequence	Previous amino acid	Next amino acid	Peptide identification probability		SEQUEST XCorr score	SEQUEST DCn score
	Modifications identified by spectrum		Actual peptide mass (AMU)		Spectrum charge	Actual minus calculated peptide mass (AMU)			Actual minus calculated peptide mass (PPM)			Peptide start index	Peptide stop index	
3369-Seal_20percent_Condensed		Uncategorized Sample		Bio Sample 1	Seal_20percent	recombinant_protein_20130729		GAV-CpCWB	recombinant_protein_20130729		100.0%	1.61%	26,812.4	12
	29	97	Seal_20percent.221.221.2		AVGEAHAR	K	G	99.0%	1.95	0.359		809.41	2	-0.0017
	-2.1	165	172											
3369-Seal_20percent_Condensed		Uncategorized Sample		Bio Sample 1	Seal_20percent	recombinant_protein_20130729		GAV-CpCWB	recombinant_protein_20130729		100.0%	1.61%	26,812.4	12
	29	97	Seal_20percent.2895.2895.2		EGDQSLTLDQR	R	A	99.7%	2.68	0.325		1,260.59	2	-0.0027
	-2.2	53	63											
3369-Seal_20percent_Condensed		Uncategorized Sample		Bio Sample 1	Seal_20percent	recombinant_protein_20130729		GAV-CpCWB	recombinant_protein_20130729		100.0%	1.61%	26,812.4	12
	29	97	Seal_20percent.2549.2549.2		EGDQSLTLDQR	R	A	99.7%	2.88	0.326		1,260.59	2	-0.0031
	-2.5	53	63											
3369-Seal_20percent_Condensed		Uncategorized Sample		Bio Sample 1	Seal_20percent	recombinant_protein_20130729		GAV-CpCWB	recombinant_protein_20130729		100.0%	1.61%	26,812.4	12
	29	97	Seal_20percent.2277.2277.3		GHGGSDPGAVANGLQEK		K	N	99.7%	3.21	0.298		1,592.75	3
	-0.0038	-2.4	9	25										
3369-Seal_20percent_Condensed		Uncategorized Sample		Bio Sample 1	Seal_20percent	recombinant_protein_20130729		GAV-CpCWB	recombinant_protein_20130729		100.0%	1.61%	26,812.4	12
	29	97	Seal_20percent.2272.2272.2		GHGGSDPGAVANGLQEK		K	N	99.7%	3.08	0.601		1,592.75	2
	-0.0020	-1.3	9	25										
3369-Seal_20percent_Condensed		Uncategorized Sample		Bio Sample 1	Seal_20percent	recombinant_protein_20130729		GAV-CpCWB	recombinant_protein_20130729		100.0%	1.61%	26,812.4	12
	29	97	Seal_20percent.9218.9218.4		GTGFEIYVHPNASPQSIALQNVLHGEILSAMR		K	Q	99.7%	2.50	0.317	Oxidation (+16)	3,464.74	4
	-0.013	-3.8	85	116										
3369-Seal_20percent_Condensed		Uncategorized Sample		Bio Sample 1	Seal_20percent	recombinant_protein_20130729		GAV-CpCWB	recombinant_protein_20130729		100.0%	1.61%	26,812.4	12
	29	97	Seal_20percent.8558.8558.3		GTGFEIYVHPNASPQSIALQNVLHGEILSAMR		K	Q	99.7%	2.38	0.339	Oxidation (+16)	3,464.74	3
	-0.0093	-2.7	85	116										
3369-Seal_20percent_Condensed		Uncategorized Sample		Bio Sample 1	Seal_20percent	recombinant_protein_20130729		GAV-CpCWB	recombinant_protein_20130729		100.0%	1.61%	26,812.4	12
	29	97	Seal_20percent.8598.8598.5		GTGFEIYVHPNASPQSIALQNVLHGEILSAMR		K	Q	99.7%	3.18	0.293	Oxidation (+16)	3,464.75	5
	0.00024	0.068	85	116										
3369-Seal_20percent_Condensed		Uncategorized Sample		Bio Sample 1	Seal_20percent	recombinant_protein_20130729		GAV-CpCWB	recombinant_protein_20130729		100.0%	1.61%	26,812.4	12
	29	97	Seal_20percent.8845.8845.4		GTGFEIYVHPNASPQSIALQNVLHGEILSAMR		K	Q	99.7%	3.37	0.308	Oxidation (+16)	3,465.73	4
	0.98	280	85	116										
3369-Seal_20percent_Condensed		Uncategorized Sample		Bio Sample 1	Seal_20percent	recombinant_protein_20130729		GAV-CpCWB	recombinant_protein_20130729		100.0%	1.61%	26,812.4	12
	29	97	Seal_20percent.8936.8936.4		GTGFEIYVHPNASPQSIALQNVLHGEILSAMR		K	Q	99.7%	2.55	0.340	Oxidation (+16)	3,464.74	4
	-0.0079	-2.3	85	116										
3369-Seal_20percent_Condensed		Uncategorized Sample		Bio Sample 1	Seal_20percent	recombinant_protein_20130729		GAV-CpCWB	recombinant_protein_20130729		100.0%	1.61%	26,812.4	12
	29	97	Seal_20percent.9587.9587.4		GTGFEIYVHPNASPQSIALQNVLHGEILSAMR		K	Q	99.7%	2.76	0.353		3,448.75	4
	-0.0081	-2.4	85	116										
3369-Seal_20percent_Condensed		Uncategorized Sample		Bio Sample 1	Seal_20percent	recombinant_protein_20130729		GAV-CpCWB	recombinant_protein_20130729		100.0%	1.61%	26,812.4	12
	29	97	Seal_20percent.9074.9074.4		GTGFEIYVHPNASPQSIALQNVLHGEILSAMR		K	Q	99.7%	2.85	0.373	Oxidation (+16)	3,464.74	4
	-0.016	-4.7	85	116										
3369-Seal_20percent_Condensed		Uncategorized Sample		Bio Sample 1	Seal_20percent	recombinant_protein_20130729		GAV-CpCWB	recombinant_protein_20130729		100.0%	1.61%	26,812.4	12
	29	97	Seal_20percent.9430.9430.4		GTGFEIYVHPNASPQSIALQNVLHGEILSAMR		K	Q	99.7%	3.64	0.371		3,448.75	4
	-0.0030	-0.87	85	116										
3369-Seal_20percent_Condensed		Uncategorized Sample		Bio Sample 1	Seal_20percent	recombinant_protein_20130729		GAV-CpCWB	recombinant_protein_20130729		100.0%	1.61%	26,812.4	12
	29	97	Seal_20percent.8987.8987.4		GTGFEIYVHPNASPQSIALQNVLHGEILSAMR		K	Q	99.7%	3.37	0.457	Oxidation (+16)	3,465.73	4
	0.98	280	85	116										
3369-Seal_20percent_Condensed		Uncategorized Sample		Bio Sample 1	Seal_20percent	recombinant_protein_20130729		GAV-CpCWB	recombinant_protein_20130729		100.0%	1.61%	26,812.4	12
	29	97	Seal_20percent.9650.9650.4		GTGFEIYVHPNASPQSIALQNVLHGEILSAMR		K	Q	99.7%	3.99	0.456		3,449.74	4
	0.98	280	85	116										
3369-Seal_20percent_Condensed		Uncategorized Sample		Bio Sample 1	Seal_20percent	recombinant_protein_20130729		GAV-CpCWB	recombinant_protein_20130729		100.0%	1.61%	26,812.4	12
	29	97	Seal_20percent.9641.9641.3		GTGFEIYVHPNASPQSIALQNVLHGEILSAMR		K	Q	99.7%	3.78	0.467		3,449.73	3
	0.98	280	85	116										
3369-Seal_20percent_Condensed		Uncategorized Sample		Bio Sample 1	Seal_20percent	recombinant_protein_20130729		GAV-CpCWB	recombinant_protein_20130729		100.0%	1.61%	26,812.4	12
	29	97	Seal_20percent.8847.8847.3		GTGFEIYVHPNASPQSIALQNVLHGEILSAMR		K	Q	99.7%	3.10	0.507	Oxidation (+16)	3,465.74	3
	0.98	280	85	116										
3369-Seal_20percent_Condensed		Uncategorized Sample		Bio Sample 1	Seal_20percent	recombinant_protein_20130729		GAV-CpCWB	recombinant_protein_20130729		100.0%	1.61%	26,812.4	12
	29	97	Seal_20percent.9364.9364.5		GTGFEIYVHPNASPQSIALQNVLHGEILSAMR		K	Q	99.7%	3.73	0.449		3,448.74	5
	-0.020	-5.8	85	116										
3369-Seal_20percent_Condensed		Uncategorized Sample		Bio Sample 1	Seal_20percent	recombinant_protein_20130729		GAV-CpCWB	recombinant_protein_20130729		100.0%	1.61%	26,812.4	12
	29	97	Seal_20percent.9316.9316.3		GTGFEIYVHPNASPQSIALQNVLHGEILSAMR		K	Q	99.7%	3.76	0.449		3,448.76	3
	0.0038	1.1	85	116										
3369-Seal_20percent_Condensed		Uncategorized Sample		Bio Sample 1	Seal_20percent	recombinant_protein_20130729		GAV-CpCWB	recombinant_protein_20130729		100.0%	1.61%	26,812.4	12
	29	97	Seal_20percent.9453.9453.3		GTGFEIYVHPNASPQSIALQNVLHGEILSAMR		K	Q	99.7%	4.41	0.493		3,448.76	3
	-0.00098	-0.28	85	116										
3369-Seal_20percent_Condensed		Uncategorized Sample		Bio Sample 1	Seal_20percent	recombinant_protein_20130729		GAV-CpCWB	recombinant_protein_20130729		100.0%	1.61%	26,812.4	12
	29	97	Seal_20percent.8656.8656.4		GTGFEIYVHPNASPQSIALQNVLHGEILSAMR		K	Q	99.7%	4.93	0.559	Oxidation (+16)	3,464.74	4
	-0.0065	-1.9	85	116										
3369-Seal_20percent_Condensed		Uncategorized Sample		Bio Sample 1	Seal_20percent	recombinant_protein_20130729		GAV-CpCWB	recombinant_protein_20130729		100.0%	1.61%	26,812.4	12
	29	97	Seal_20percent.4527.4527.4		IFWDKGHGGSDPGAVANGLQEK		K	N	99.7%	3.01	0.275		2,282.10	4
	-0.0073	-3.2	4	25										
3369-Seal_20percent_Condensed		Uncategorized Sample		Bio Sample 1	Seal_20percent	recombinant_protein_20130729		GAV-CpCWB	recombinant_protein_20130729		100.0%	1.61%	26,812.4	12
	29	97	Seal_20percent.4530.4530.3		IFWDKGHGGSDPGAVANGLQEK		K	N	99.7%	3.35	0.314		2,282.10	3
	-0.0038	-1.7	4	25										
3369-Seal_20percent_Condensed		Uncategorized Sample		Bio Sample 1	Seal_20percent	recombinant_protein_20130729		GAV-CpCWB	recombinant_protein_20130729		100.0%	1.61%	26,812.4	12
	29	97	Seal_20percent.4564.4564.2		IFWDKGHGGSDPGAVANGLQEK		K	N	99.7%	2.49	0.494		2,282.11	2
	0.0018	0.80	4	25										
3369-Seal_20percent_Condensed		Uncategorized Sample		Bio Sample 1	Seal_20percent	recombinant_protein_20130729		GAV-CpCWB	recombinant_protein_20130729		100.0%	1.61%	26,812.4	12
	29	97	Seal_20percent.6429.6429.2		ISNELGYYCFAEK	R	I	99.7%	2.36	0.396	Carbamidomethyl (+57)		1,592.72	2
	0.0017	1.1	219	231										

3369-Seal_20percent_Condensed		Uncategorized Sample		Bio Sample 1	Seal_20percent	recombinant_protein_20130729		GAV-CpCWB	recombinant_protein_20130729		100.0%	1.61%	26,812.4	12
	29	97	Seal_20percent.5382.5382.2		ISNELGYYCFAEK	R	I	99.7%	2.35	0.438	Carbamidomethyl (+57)		1,592.71	2
	-0.0049	-3.1	219	231										
3369-Seal_20percent_Condensed		Uncategorized Sample		Bio Sample 1	Seal_20percent	recombinant_protein_20130729		GAV-CpCWB	recombinant_protein_20130729		100.0%	1.61%	26,812.4	12
	29	97	Seal_20percent.6289.6289.2		ISNELGYYCFAEK	R	I	99.7%	2.73	0.467	Carbamidomethyl (+57)		1,592.72	2
	0.0013	0.83	219	231										
3369-Seal_20percent_Condensed		Uncategorized Sample		Bio Sample 1	Seal_20percent	recombinant_protein_20130729		GAV-CpCWB	recombinant_protein_20130729		100.0%	1.61%	26,812.4	12
	29	97	Seal_20percent.5708.5708.2		ISNELGYYCFAEK	R	I	99.7%	4.05	0.392	Carbamidomethyl (+57)		1,593.71	2
	0.99	620	219	231										
3369-Seal_20percent_Condensed		Uncategorized Sample		Bio Sample 1	Seal_20percent	recombinant_protein_20130729		GAV-CpCWB	recombinant_protein_20130729		100.0%	1.61%	26,812.4	12
	29	97	Seal_20percent.6154.6154.2		ISNELGYYCFAEK	R	I	99.7%	3.06	0.451	Carbamidomethyl (+57)		1,592.72	2
	0.0023	1.4	219	231										
3369-Seal_20percent_Condensed		Uncategorized Sample		Bio Sample 1	Seal_20percent	recombinant_protein_20130729		GAV-CpCWB	recombinant_protein_20130729		100.0%	1.61%	26,812.4	12
	29	97	Seal_20percent.5860.5860.2		ISNELGYYCFAEK	R	I	99.7%	3.99	0.443	Carbamidomethyl (+57)		1,592.73	2
	0.0097	6.1	219	231										
3369-Seal_20percent_Condensed		Uncategorized Sample		Bio Sample 1	Seal_20percent	recombinant_protein_20130729		GAV-CpCWB	recombinant_protein_20130729		100.0%	1.61%	26,812.4	12
	29	97	Seal_20percent.5497.5497.2		ISNELGYYCFAEK	R	I	99.7%	4.39	0.424	Carbamidomethyl (+57)		1,592.71	2
	-0.0037	-2.3	219	231										
3369-Seal_20percent_Condensed		Uncategorized Sample		Bio Sample 1	Seal_20percent	recombinant_protein_20130729		GAV-CpCWB	recombinant_protein_20130729		100.0%	1.61%	26,812.4	12
	29	97	Seal_20percent.6191.6191.2		ISNELGYYCFAEK	R	I	99.7%	4.35	0.520	Carbamidomethyl (+57)		1,591.72	2
	-1.00	-630	219	231										
3369-Seal_20percent_Condensed		Uncategorized Sample		Bio Sample 1	Seal_20percent	recombinant_protein_20130729		GAV-CpCWB	recombinant_protein_20130729		100.0%	1.61%	26,812.4	12
	29	97	Seal_20percent.5993.5993.2		ISNELGYYCFAEK	R	I	99.7%	3.45	0.566	Carbamidomethyl (+57)		1,592.72	2
	0.0022	1.4	219	231										
3369-Seal_20percent_Condensed		Uncategorized Sample		Bio Sample 1	Seal_20percent	recombinant_protein_20130729		GAV-CpCWB	recombinant_protein_20130729		100.0%	1.61%	26,812.4	12
	29	97	Seal_20percent.5647.5647.2		ISNELGYYCFAEK	R	I	99.7%	4.72	0.478	Carbamidomethyl (+57)		1,592.71	2
	-0.0032	-2.0	219	231										
3369-Seal_20percent_Condensed		Uncategorized Sample		Bio Sample 1	Seal_20percent	recombinant_protein_20130729		GAV-CpCWB	recombinant_protein_20130729		100.0%	1.61%	26,812.4	12
	29	97	Seal_20percent.5573.5573.3		ISNELGYYCFAEK	R	I	99.7%	3.99	0.500	Carbamidomethyl (+57)		1,592.71	3
	-0.0040	-2.5	219	231										
3369-Seal_20percent_Condensed		Uncategorized Sample		Bio Sample 1	Seal_20percent	recombinant_protein_20130729		GAV-CpCWB	recombinant_protein_20130729		100.0%	1.61%	26,812.4	12
	29	97	Seal_20percent.5433.5433.3		ISNELGYYCFAEK	R	I	99.7%	3.91	0.547	Carbamidomethyl (+57)		1,592.72	3
	-0.0029	-1.8	219	231										
3369-Seal_20percent_Condensed		Uncategorized Sample		Bio Sample 1	Seal_20percent	recombinant_protein_20130729		GAV-CpCWB	recombinant_protein_20130729		100.0%	1.61%	26,812.4	12
	29	97	Seal_20percent.7247.7247.2		IVEYATDYLAAHYEGFTQR		K	V	99.7%	2.88	0.378		2,246.05	2
	-0.017	-7.6	31	49										
3369-Seal_20percent_Condensed		Uncategorized Sample		Bio Sample 1	Seal_20percent	recombinant_protein_20130729		GAV-CpCWB	recombinant_protein_20130729		100.0%	1.61%	26,812.4	12
	29	97	Seal_20percent.8282.8282.3		IVEYATDYLAAHYEGFTQR		K	V	99.7%	1.95	0.444		2,246.06	3
	-0.0066	-2.9	31	49										
3369-Seal_20percent_Condensed		Uncategorized Sample		Bio Sample 1	Seal_20percent	recombinant_protein_20130729		GAV-CpCWB	recombinant_protein_20130729		100.0%	1.61%	26,812.4	12
	29	97	Seal_20percent.5913.5913.2		IVEYATDYLAAHYEGFTQR		K	V	99.7%	2.86	0.516		2,246.06	2
	-0.0024	-1.1	31	49										
3369-Seal_20percent_Condensed		Uncategorized Sample		Bio Sample 1	Seal_20percent	recombinant_protein_20130729		GAV-CpCWB	recombinant_protein_20130729		100.0%	1.61%	26,812.4	12
	29	97	Seal_20percent.7126.7126.2		IVEYATDYLAAHYEGFTQR		K	V	99.7%	2.55	0.556		2,246.06	2
	0.000079	0.035	31	49										
3369-Seal_20percent_Condensed		Uncategorized Sample		Bio Sample 1	Seal_20percent	recombinant_protein_20130729		GAV-CpCWB	recombinant_protein_20130729		100.0%	1.61%	26,812.4	12
	29	97	Seal_20percent.6788.6788.3		IVEYATDYLAAHYEGFTQR		K	V	99.7%	2.99	0.410		2,246.06	3
	-0.0066	-2.9	31	49										
3369-Seal_20percent_Condensed		Uncategorized Sample		Bio Sample 1	Seal_20percent	recombinant_protein_20130729		GAV-CpCWB	recombinant_protein_20130729		100.0%	1.61%	26,812.4	12
	29	97	Seal_20percent.7312.7312.3		IVEYATDYLAAHYEGFTQR		K	V	99.7%	2.28	0.479		2,246.06	3
	-0.0031	-1.4	31	49										
3369-Seal_20percent_Condensed		Uncategorized Sample		Bio Sample 1	Seal_20percent	recombinant_protein_20130729		GAV-CpCWB	recombinant_protein_20130729		100.0%	1.61%	26,812.4	12
	29	97	Seal_20percent.7185.7185.3		IVEYATDYLAAHYEGFTQR		K	V	99.7%	3.68	0.383		2,246.06	3
	-0.0046	-2.0	31	49										
3369-Seal_20percent_Condensed		Uncategorized Sample		Bio Sample 1	Seal_20percent	recombinant_protein_20130729		GAV-CpCWB	recombinant_protein_20130729		100.0%	1.61%	26,812.4	12
	29	97	Seal_20percent.6794.6794.2		IVEYATDYLAAHYEGFTQR		K	V	99.7%	3.26	0.523		2,246.06	2
	-0.0055	-2.5	31	49										
3369-Seal_20percent_Condensed		Uncategorized Sample		Bio Sample 1	Seal_20percent	recombinant_protein_20130729		GAV-CpCWB	recombinant_protein_20130729		100.0%	1.61%	26,812.4	12
	29	97	Seal_20percent.8149.8149.3		IVEYATDYLAAHYEGFTQR		K	V	99.7%	2.50	0.472		2,246.06	3
	-0.0055	-2.4	31	49										
3369-Seal_20percent_Condensed		Uncategorized Sample		Bio Sample 1	Seal_20percent	recombinant_protein_20130729		GAV-CpCWB	recombinant_protein_20130729		100.0%	1.61%	26,812.4	12
	29	97	Seal_20percent.6294.6294.2		IVEYATDYLAAHYEGFTQR		K	V	99.7%	3.07	0.598		2,246.06	2
	-0.0065	-2.9	31	49										
3369-Seal_20percent_Condensed		Uncategorized Sample		Bio Sample 1	Seal_20percent	recombinant_protein_20130729		GAV-CpCWB	recombinant_protein_20130729		100.0%	1.61%	26,812.4	12
	29	97	Seal_20percent.6947.6947.4		IVEYATDYLAAHYEGFTQR		K	V	99.7%	4.01	0.428		2,246.06	4
	-0.0057	-2.5	31	49										
3369-Seal_20percent_Condensed		Uncategorized Sample		Bio Sample 1	Seal_20percent	recombinant_protein_20130729		GAV-CpCWB	recombinant_protein_20130729		100.0%	1.61%	26,812.4	12
	29	97	Seal_20percent.6997.6997.2		IVEYATDYLAAHYEGFTQR		K	V	99.7%	4.00	0.585		2,246.06	2
	-0.0068	-3.0	31	49										
3369-Seal_20percent_Condensed		Uncategorized Sample		Bio Sample 1	Seal_20percent	recombinant_protein_20130729		GAV-CpCWB	recombinant_protein_20130729		100.0%	1.61%	26,812.4	12
	29	97	Seal_20percent.7063.7063.3		IVEYATDYLAAHYEGFTQR		K	V	99.7%	2.97	0.518		2,246.06	3
	-0.0027	-1.2	31	49										
3369-Seal_20percent_Condensed		Uncategorized Sample		Bio Sample 1	Seal_20percent	recombinant_protein_20130729		GAV-CpCWB	recombinant_protein_20130729		100.0%	1.61%	26,812.4	12
	29	97	Seal_20percent.6802.6802.4		IVEYATDYLAAHYEGFTQR		K	V	99.7%	4.25	0.480		2,246.06	4
	-0.0013	-0.57	31	49										
3369-Seal_20percent_Condensed		Uncategorized Sample		Bio Sample 1	Seal_20percent	recombinant_protein_20130729		GAV-CpCWB	recombinant_protein_20130729		100.0%	1.61%	26,812.4	12
	29	97	Seal_20percent.6311.6311.3		IVEYATDYLAAHYEGFTQR		K	V	99.7%	3.70	0.595		2,246.06	3
	-0.0024	-1.1	31	49										

3369-Seal_20percent_Condensed		Uncategorized Sample		Bio Sample 1	Seal_20percent	recombinant_protein_20130729		GAV-CpCWB	recombinant_protein_20130729		100.0%	1.61%	26,812.4	12
	29	97	Seal_20percent.6924.6924.3		IVEYATDYLAAHYEGFTQR		K	V	99.7%	3.78	0.601		2,246.06	3
	-0.0024	-1.1	31	49										
3369-Seal_20percent_Condensed		Uncategorized Sample		Bio Sample 1	Seal_20percent	recombinant_protein_20130729		GAV-CpCWB	recombinant_protein_20130729		100.0%	1.61%	26,812.4	12
	29	97	Seal_20percent.6351.6351.2		MPAVLTENLFIDSNDAK		K	H	99.7%	1.88	0.398	Oxidation (+16)	1,892.91	2
	-0.0065	-3.4	139	155										
3369-Seal_20percent_Condensed		Uncategorized Sample		Bio Sample 1	Seal_20percent	recombinant_protein_20130729		GAV-CpCWB	recombinant_protein_20130729		100.0%	1.61%	26,812.4	12
	29	97	Seal_20percent.6636.6636.3		MPAVLTENLFIDSNDAK		K	H	99.7%	3.99	0.156	Oxidation (+16)	1,892.92	3
	-0.0038	-2.0	139	155										
3369-Seal_20percent_Condensed		Uncategorized Sample		Bio Sample 1	Seal_20percent	recombinant_protein_20130729		GAV-CpCWB	recombinant_protein_20130729		100.0%	1.61%	26,812.4	12
	29	97	Seal_20percent.6189.6189.2		MPAVLTENLFIDSNDAK		K	H	99.7%	2.32	0.334	Oxidation (+16)	1,892.92	2
	0.0021	1.1	139	155										
3369-Seal_20percent_Condensed		Uncategorized Sample		Bio Sample 1	Seal_20percent	recombinant_protein_20130729		GAV-CpCWB	recombinant_protein_20130729		100.0%	1.61%	26,812.4	12
	29	97	Seal_20percent.7332.7332.2		MPAVLTENLFIDSNDAK		K	H	99.7%	2.87	0.289	Oxidation (+16)	1,892.91	2
	-0.0051	-2.7	139	155										
3369-Seal_20percent_Condensed		Uncategorized Sample		Bio Sample 1	Seal_20percent	recombinant_protein_20130729		GAV-CpCWB	recombinant_protein_20130729		100.0%	1.61%	26,812.4	12
	29	97	Seal_20percent.7399.7399.2		MPAVLTENLFIDSNDAK		K	H	99.7%	2.85	0.396		1,877.91	2
	0.99	530	139	155										
3369-Seal_20percent_Condensed		Uncategorized Sample		Bio Sample 1	Seal_20percent	recombinant_protein_20130729		GAV-CpCWB	recombinant_protein_20130729		100.0%	1.61%	26,812.4	12
	29	97	Seal_20percent.7870.7870.3		MPAVLTENLFIDSNDAK		K	H	99.7%	3.72	0.235		1,876.92	3
	-0.0076	-4.0	139	155										
3369-Seal_20percent_Condensed		Uncategorized Sample		Bio Sample 1	Seal_20percent	recombinant_protein_20130729		GAV-CpCWB	recombinant_protein_20130729		100.0%	1.61%	26,812.4	12
	29	97	Seal_20percent.7592.7592.3		MPAVLTENLFIDSNDAK		K	H	99.7%	3.94	0.228		1,876.92	3
	-0.0063	-3.3	139	155										
3369-Seal_20percent_Condensed		Uncategorized Sample		Bio Sample 1	Seal_20percent	recombinant_protein_20130729		GAV-CpCWB	recombinant_protein_20130729		100.0%	1.61%	26,812.4	12
	29	97	Seal_20percent.8099.8099.2		MPAVLTENLFIDSNDAK		K	H	99.7%	3.11	0.345		1,876.92	2
	-0.0030	-1.6	139	155										
3369-Seal_20percent_Condensed		Uncategorized Sample		Bio Sample 1	Seal_20percent	recombinant_protein_20130729		GAV-CpCWB	recombinant_protein_20130729		100.0%	1.61%	26,812.4	12
	29	97	Seal_20percent.7471.7471.2		MPAVLTENLFIDSNDAK		K	H	99.7%	2.96	0.398	Oxidation (+16)	1,892.92	2
	-0.0032	-1.7	139	155										
3369-Seal_20percent_Condensed		Uncategorized Sample		Bio Sample 1	Seal_20percent	recombinant_protein_20130729		GAV-CpCWB	recombinant_protein_20130729		100.0%	1.61%	26,812.4	12
	29	97	Seal_20percent.6776.6776.3		MPAVLTENLFIDSNDAK		K	H	99.7%	3.94	0.267	Oxidation (+16)	1,892.92	3
	-0.0033	-1.7	139	155										
3369-Seal_20percent_Condensed		Uncategorized Sample		Bio Sample 1	Seal_20percent	recombinant_protein_20130729		GAV-CpCWB	recombinant_protein_20130729		100.0%	1.61%	26,812.4	12
	29	97	Seal_20percent.7177.7177.2		MPAVLTENLFIDSNDAK		K	H	99.7%	2.62	0.472		1,876.92	2
	-0.0017	-0.90	139	155										
3369-Seal_20percent_Condensed		Uncategorized Sample		Bio Sample 1	Seal_20percent	recombinant_protein_20130729		GAV-CpCWB	recombinant_protein_20130729		100.0%	1.61%	26,812.4	12
	29	97	Seal_20percent.8225.8225.2		MPAVLTENLFIDSNDAK		K	H	99.7%	2.40	0.505		1,876.92	2
	-0.0032	-1.7	139	155										
3369-Seal_20percent_Condensed		Uncategorized Sample		Bio Sample 1	Seal_20percent	recombinant_protein_20130729		GAV-CpCWB	recombinant_protein_20130729		100.0%	1.61%	26,812.4	12
	29	97	Seal_20percent.7386.7386.2		MPAVLTENLFIDSNDAK		K	H	99.7%	2.78	0.517		1,876.92	2
	-0.0021	-1.1	139	155										
3369-Seal_20percent_Condensed		Uncategorized Sample		Bio Sample 1	Seal_20percent	recombinant_protein_20130729		GAV-CpCWB	recombinant_protein_20130729		100.0%	1.61%	26,812.4	12
	29	97	Seal_20percent.6945.6945.2		MPAVLTENLFIDSNDAK		K	H	99.7%	4.43	0.429	Oxidation (+16)	1,892.92	2
	-0.0019	-0.98	139	155										
3369-Seal_20percent_Condensed		Uncategorized Sample		Bio Sample 1	Seal_20percent	recombinant_protein_20130729		GAV-CpCWB	recombinant_protein_20130729		100.0%	1.61%	26,812.4	12
	29	97	Seal_20percent.6482.6482.2		MPAVLTENLFIDSNDAK		K	H	99.7%	3.63	0.502	Oxidation (+16)	1,892.91	2
	-0.0065	-3.4	139	155										
3369-Seal_20percent_Condensed		Uncategorized Sample		Bio Sample 1	Seal_20percent	recombinant_protein_20130729		GAV-CpCWB	recombinant_protein_20130729		100.0%	1.61%	26,812.4	12
	29	97	Seal_20percent.7601.7601.2		MPAVLTENLFIDSNDAK		K	H	99.7%	4.08	0.498	Oxidation (+16)	1,892.92	2
	0.000100	0.053	139	155										
3369-Seal_20percent_Condensed		Uncategorized Sample		Bio Sample 1	Seal_20percent	recombinant_protein_20130729		GAV-CpCWB	recombinant_protein_20130729		100.0%	1.61%	26,812.4	12
	29	97	Seal_20percent.7518.7518.2		MPAVLTENLFIDSNDAK		K	H	99.7%	4.48	0.480		1,876.92	2
	-0.0017	-0.90	139	155										
3369-Seal_20percent_Condensed		Uncategorized Sample		Bio Sample 1	Seal_20percent	recombinant_protein_20130729		GAV-CpCWB	recombinant_protein_20130729		100.0%	1.61%	26,812.4	12
	29	97	Seal_20percent.7709.7709.3		MPAVLTENLFIDSNDAK		K	H	99.7%	5.04	0.365		1,876.92	3
	-0.0055	-3.0	139	155										
3369-Seal_20percent_Condensed		Uncategorized Sample		Bio Sample 1	Seal_20percent	recombinant_protein_20130729		GAV-CpCWB	recombinant_protein_20130729		100.0%	1.61%	26,812.4	12
	29	97	Seal_20percent.6874.6874.2		MPAVLTENLFIDSNDAK		K	H	99.7%	4.72	0.527	Oxidation (+16)	1,893.91	2
	0.99	520	139	155										
3369-Seal_20percent_Condensed		Uncategorized Sample		Bio Sample 1	Seal_20percent	recombinant_protein_20130729		GAV-CpCWB	recombinant_protein_20130729		100.0%	1.61%	26,812.4	12
	29	97	Seal_20percent.7728.7728.2		MPAVLTENLFIDSNDAK		K	H	99.7%	3.76	0.566	Oxidation (+16)	1,892.91	2
	-0.0050	-2.7	139	155										
3369-Seal_20percent_Condensed		Uncategorized Sample		Bio Sample 1	Seal_20percent	recombinant_protein_20130729		GAV-CpCWB	recombinant_protein_20130729		100.0%	1.61%	26,812.4	12
	29	97	Seal_20percent.7076.7076.2		MPAVLTENLFIDSNDAK		K	H	99.7%	4.10	0.538	Oxidation (+16)	1,892.92	2
	0.0051	2.7	139	155										
3369-Seal_20percent_Condensed		Uncategorized Sample		Bio Sample 1	Seal_20percent	recombinant_protein_20130729		GAV-CpCWB	recombinant_protein_20130729		100.0%	1.61%	26,812.4	12
	29	97	Seal_20percent.7200.7200.2		MPAVLTENLFIDSNDAK		K	H	99.7%	4.05	0.556	Oxidation (+16)	1,892.92	2
	-0.0034	-1.8	139	155										
3369-Seal_20percent_Condensed		Uncategorized Sample		Bio Sample 1	Seal_20percent	recombinant_protein_20130729		GAV-CpCWB	recombinant_protein_20130729		100.0%	1.61%	26,812.4	12
	29	97	Seal_20percent.7781.7781.2		MPAVLTENLFIDSNDAK		K	H	99.7%	5.90	0.500		1,877.91	2
	0.99	530	139	155										
3369-Seal_20percent_Condensed		Uncategorized Sample		Bio Sample 1	Seal_20percent	recombinant_protein_20130729		GAV-CpCWB	recombinant_protein_20130729		100.0%	1.61%	26,812.4	12
	29	97	Seal_20percent.7869.7869.2		MPAVLTENLFIDSNDAK		K	H	99.7%	5.13	0.519		1,876.92	2
	-0.0043	-2.3	139	155										
3369-Seal_20percent_Condensed		Uncategorized Sample		Bio Sample 1	Seal_20percent	recombinant_protein_20130729		GAV-CpCWB	recombinant_protein_20130729		100.0%	1.61%	26,812.4	12
	29	97	Seal_20percent.6805.6805.2		MPAVLTENLFIDSNDAK		K	H	99.7%	5.17	0.549	Oxidation (+16)	1,892.92	2
	-0.0020	-1.0	139	155										

3369-Seal_20percent_Condensed		Uncategorized Sample		Bio Sample 1	Seal_20percent	recombinant_protein_20130729		GAV-CpCWB	recombinant_protein_20130729		100.0%	1.61%	26,812.4	12
	29	97	Seal_20percent.6619.6619.2		MPAVLTENLFIDSNDAK		K	H	99.7%	5.63	0.527	Oxidation (+16)	1,892.92	2
	-0.0022	-1.2	139	155										
3369-Seal_20percent_Condensed		Uncategorized Sample		Bio Sample 1	Seal_20percent	recombinant_protein_20130729		GAV-CpCWB	recombinant_protein_20130729		100.0%	1.61%	26,812.4	12
	29	97	Seal_20percent.7983.7983.2		MPAVLTENLFIDSNDAK		K	H	99.7%	4.65	0.605		1,876.92	2
	-0.0013	-0.70	139	155										
3369-Seal_20percent_Condensed		Uncategorized Sample		Bio Sample 1	Seal_20percent	recombinant_protein_20130729		GAV-CpCWB	recombinant_protein_20130729		100.0%	1.61%	26,812.4	12
	29	97	Seal_20percent.7658.7658.2		MPAVLTENLFIDSNDAK		K	H	99.7%	5.63	0.600		1,876.92	2
	-0.0019	-1.0	139	155										
3369-Seal_20percent_Condensed		Uncategorized Sample		Bio Sample 1	Seal_20percent	recombinant_protein_20130729		GAV-CpCWB	recombinant_protein_20130729		100.0%	1.61%	26,812.4	12
	29	97	Seal_20percent.3760.3760.3		NEAFLKAVGEAHAR	K	G	99.7%	1.96	0.364		1,511.78	3	-0.0032
	-2.1	159	172											
3369-Seal_20percent_Condensed		Uncategorized Sample		Bio Sample 1	Seal_20percent	recombinant_protein_20130729		GAV-CpCWB	recombinant_protein_20130729		100.0%	1.61%	26,812.4	12
	29	97	Seal_20percent.9195.9195.4		NLTHKIVEYATDYLAAHYEGFTQR		K	V	99.7%	3.06	0.212		2,839.39	4
	-0.0049	-1.7	26	49										
3369-Seal_20percent_Condensed		Uncategorized Sample		Bio Sample 1	Seal_20percent	recombinant_protein_20130729		GAV-CpCWB	recombinant_protein_20130729		100.0%	1.61%	26,812.4	12
	29	97	Seal_20percent.9334.9334.4		NLTHKIVEYATDYLAAHYEGFTQR		K	V	99.7%	4.03	0.539		2,839.37	4
	-0.018	-6.4	26	49										
3369-Seal_20percent_Condensed		Uncategorized Sample		Bio Sample 1	Seal_20percent	recombinant_protein_20130729		GAV-CpCWB	recombinant_protein_20130729		100.0%	1.61%	26,812.4	12
	29	97	Seal_20percent.9247.9247.3		NLTHKIVEYATDYLAAHYEGFTQR		K	V	99.7%	3.80	0.564		2,839.40	3
	0.0045	1.6	26	49										
3369-Seal_20percent_Condensed		Uncategorized Sample		Bio Sample 1	Seal_20percent	recombinant_protein_20130729		GAV-CpCWB	recombinant_protein_20130729		100.0%	1.61%	26,812.4	12
	29	97	Seal_20percent.6645.6645.2		YLANAIDPNIPLEK	R	E	99.7%	2.78	0.256		1,569.84	2	0.00057
	0.36	181	194											
3369-Seal_20percent_Condensed		Uncategorized Sample		Bio Sample 1	Seal_20percent	recombinant_protein_20130729		GAV-CpCWB	recombinant_protein_20130729		100.0%	1.61%	26,812.4	12
	29	97	Seal_20percent.5875.5875.3		YLANAIDPNIPLEK	R	E	99.7%	3.78	0.224		1,569.84	3	-0.0034
	-2.1	181	194											
3369-Seal_20percent_Condensed		Uncategorized Sample		Bio Sample 1	Seal_20percent	recombinant_protein_20130729		GAV-CpCWB	recombinant_protein_20130729		100.0%	1.61%	26,812.4	12
	29	97	Seal_20percent.6783.6783.2		YLANAIDPNIPLEK	R	E	99.7%	2.73	0.376		1,569.84	2	-0.0024
	-1.5	181	194											
3369-Seal_20percent_Condensed		Uncategorized Sample		Bio Sample 1	Seal_20percent	recombinant_protein_20130729		GAV-CpCWB	recombinant_protein_20130729		100.0%	1.61%	26,812.4	12
	29	97	Seal_20percent.6505.6505.2		YLANAIDPNIPLEK	R	E	99.7%	3.05	0.357		1,569.83	2	-0.0072
	-4.6	181	194											
3369-Seal_20percent_Condensed		Uncategorized Sample		Bio Sample 1	Seal_20percent	recombinant_protein_20130729		GAV-CpCWB	recombinant_protein_20130729		100.0%	1.61%	26,812.4	12
	29	97	Seal_20percent.6377.6377.2		YLANAIDPNIPLEK	R	E	99.7%	3.35	0.334		1,569.84	2	-0.0029
	-1.8	181	194											
3369-Seal_20percent_Condensed		Uncategorized Sample		Bio Sample 1	Seal_20percent	recombinant_protein_20130729		GAV-CpCWB	recombinant_protein_20130729		100.0%	1.61%	26,812.4	12
	29	97	Seal_20percent.6099.6099.2		YLANAIDPNIPLEK	R	E	99.7%	3.74	0.333		1,569.84	2	-0.00090
	-0.57	181	194											
3369-Seal_20percent_Condensed		Uncategorized Sample		Bio Sample 1	Seal_20percent	recombinant_protein_20130729		GAV-CpCWB	recombinant_protein_20130729		100.0%	1.61%	26,812.4	12
	29	97	Seal_20percent.6236.6236.2		YLANAIDPNIPLEK	R	E	99.7%	3.63	0.372		1,569.84	2	-0.0025
	-1.6	181	194											
3369-Seal_20percent_Condensed		Uncategorized Sample		Bio Sample 1	Seal_20percent	recombinant_protein_20130729		GAV-CpCWB	recombinant_protein_20130729		100.0%	1.61%	26,812.4	12
	29	97	Seal_20percent.5759.5759.3		YLANAIDPNIPLEK	R	E	99.7%	3.83	0.318		1,569.84	3	-0.0041
	-2.6	181	194											
3369-Seal_20percent_Condensed		Uncategorized Sample		Bio Sample 1	Seal_20percent	recombinant_protein_20130729		GAV-CpCWB	recombinant_protein_20130729		100.0%	1.61%	26,812.4	12
	29	97	Seal_20percent.5756.5756.2		YLANAIDPNIPLEK	R	E	99.7%	4.24	0.385		1,569.84	2	-0.0025
	-1.6	181	194											
3369-Seal_20percent_Condensed		Uncategorized Sample		Bio Sample 1	Seal_20percent	recombinant_protein_20130729		GAV-CpCWB	recombinant_protein_20130729		100.0%	1.61%	26,812.4	12
	29	97	Seal_20percent.5872.5872.2		YLANAIDPNIPLEK	R	E	99.7%	4.28	0.415		1,569.84	2	-0.0027
	-1.7	181	194											
3369-Seal_20percent_Condensed		Uncategorized Sample		Bio Sample 1	Seal_20percent	recombinant_protein_20130729		GAV-CpCWB	recombinant_protein_20130729		100.0%	1.61%	26,812.4	12
	29	97	Seal_20percent.6225.6225.3		YLANAIDPNIPLEKEQDYYR		R	V	99.7%	3.12	0.257		2,424.19	3
	-0.0032	-1.3	181	200										
3369-Seal_20percent_Condensed		Uncategorized Sample		Bio Sample 1	Seal_20percent	recombinant_protein_20130729		GAV-CpCWB	recombinant_protein_20130729		100.0%	1.61%	26,812.4	12
	29	97	Seal_20percent.6230.6230.2		YLANAIDPNIPLEKEQDYYR		R	V	99.7%	2.58	0.440		2,424.19	2
	-0.0083	-3.4	181	200										
3369-Seal_20percent_Condensed		Uncategorized Sample		Bio Sample 1	Seal_20percent	recombinant_protein_20130729		GAV-CpCWB	recombinant_protein_20130729		100.0%	1.61%	26,812.4	12
	29	97	Seal_20percent.6085.6085.3		YLANAIDPNIPLEKEQDYYR		R	V	99.7%	3.39	0.384		2,424.19	3
	-0.0025	-1.0	181	200										
